# Thermoelectric Properties of Cu_2_SnSe_3_-SnS Composite

**DOI:** 10.3390/ma12132040

**Published:** 2019-06-26

**Authors:** Muhammad Siyar, Jun-Young Cho, Woo-Chan Jin, Euy Heon Hwang, Miyoung Kim, Chan Park

**Affiliations:** 1 Department of Materials Science and Engineering, Seoul National University, Seoul 08826, Korea; 2 School of Chemical and Material Engineering, NUST, H-12, Islamabad 44000, Pakistan; 3 Department of Nano Engineering, SKKU Advanced Institute of Nanotechnology, Sungkyunkwan University, Suwon 16419, Korea; 4 Research Institute of Advanced Materials, Seoul National University, Seoul 08826, Korea

**Keywords:** thermoelectric, Cu_2_SnSe_3_, composites

## Abstract

Heavily doped degenerate semiconductors such as Cu_2_SnSe_3_ (CTSe) attracted attention in thermoelectric (TE) and optoelectronic fields, due to their high electrical conductivity and small band gap. The small Seebeck coefficient of undoped CTSe, however, is the major issue in achieving high TE performance (figure of merit, ZT). Here, we report that the Seebeck coefficient of CTSe can be controlled by adding SnS within a CTSe matrix. CTSe-SnS composite has not only high Seebeck coefficient in the range of 300–500 µVolt/K but thermal conductivity which is lower than that of pristine CTSe due to the scattering at the interface between the matrix and the SnS particles. A reasonable ZT of 0.18 is achieved at 570 K by adding a small amount (3 wt.%) of SnS to the CTSe matrix.

## 1. Introduction

Thermoelectric (TE) materials are the subject of great interest for scientists and engineers, as it can directly convert waste heat into electricity [1,2]. The TE efficiency of materials is generally governed by the dimensionless figure of merit TE performance (ZT) = σS^2^T/κ, where σ is electrical conductivity, S is Seebeck coefficient, T is absolute temperature and κ is the thermal conductivity. The total thermal conductivity can be further divided into lattice thermal conductivity (κ_l_) and carrier thermal conductivity (κ_e_). A good TE material should have a large σ and S with low κ [3].

The ultimate goal of TE research is to develop TE materials with high TE performance. The improvements of ZT in chalcogenides and silicides were reported using various approaches, including nano-structuring, doping, and alloying [4,5,6]. Alloying of compounds results in disorder and lattice distortion, which enhances the phonon scattering and decreases the thermal conductivity. Optimized composition in composites can also increase the effective mass of the system, which can lead to the enhancement of Seebeck coefficient [7]. Composites, which consist of more than two phases, can lead to the formation of inclusions and interfaces, which can block the low energy phonon without affecting the electronic carrier transport significantly; hence, the low thermal conductivity is expected along with reasonable electrical conductivity [8]. This approach is an efficient way to improve TE efficiency by optimizing the electrical and thermal transport properties. Composites can also lead to point defect scattering, which can reduce the lattice thermal conductivity, and hence higher ZT can be achieved for the composite system than in a pristine compound [9].

In addition to the TE performance (ZT), the cost and environmental considerations are equally important for TE materials for their commercial large-scale applications. The existing efficient TE materials contain expensive or toxic elements such as Te, Pb, etc. [10,11]. Therefore, Cu_2_SnSe_3_ (CTSe) is a promising TE material with earth abundant, cheap, and environment-friendly elements, such as Sn and Se, which emerged in the past decade as a potential alternative for those used in TE devices today [12,13].

TE properties of CTSe are very close to those of other commercialized TE materials, i.e., PbTe, except the Seebeck coefficient which is much lower than PbTe. This leads to a ZT much lower than those of TE materials used in TE devices today. It can be expected that the increase in Seebeck coefficient along with reasonable thermal and electrical properties can lead to control of TE properties of CTSe. In this study, we report the TE properties of CTSe-SnS composites fabricated using mechanical alloying and spark plasma sintering. We successfully controlled the Seebeck coefficient by adding SnS to the CTSe matrix. Improvement of TE properties (enhanced ZT) in the medium temperature range (300–570 K) is achieved by adding 3 wt.% SnS to the CTSe matrix.

## 2. Materials and Methods

CTSe-SnS composite powder samples (0.5, 1, 3, and 5 wt.% SnS) were named according to the amount of SnS added to CTSe and synthesized by a two-step process. CTSe and SnS were prepared separately from the stoichiometric amount of precursor elements, Cu 99.99%, Sn 99.99% Se 99.99%, and S 99.99% powders (Kunjundo Chemicals) (average size 5 μm) by mechanical alloying for 5 h. Next, these two compounds were mixed and mechanical-alloyed for 1 h to obtain homogeneous composite powder samples.

Powder samples were consolidated using spark plasma sintering. An appropriate amount of powder was put into a graphite die and pressed up to 600 kgf pressure. Sintering was performed at 670 K for 10 min. The thicknesses of the consolidated samples were around 10 mm with a lateral diameter of about 12 mm. The bar samples with longitudinal direction parallel to the direction of the pressure applied during spark plasma sintering with typical dimension of 2 × 2 × 6 mm^3^ were used to measure the out-of-plane Seebeck coefficient and electrical resistivity. Phase studies were performed on the powder and pellet samples using powder X-ray diffraction (XRD) (Bruker, D-8 Advance, Berlin, Germany) with copper Kα radiation. Microstructural and energy dispersive spectroscopy (EDS) analyses were carried out using field emission-scanning electron microscope (FE-SEM) (Zeiss, Merlin Compact, Kohen, Germany). The TE properties were measured in the temperature range of 300 to 570 K, using a commercial TE property measurement system (SEEPEL, TEP-800, Gunpo-si, Korea). Laser flash technique (Netzsch, LFA 457, Germany) was used to measure the thermal diffusivity (*D*) of the carbon coated disc-shape samples with thickness <1 mm, in the temperature range of 300 to 570 K in an inert atmosphere. Thermal conductivity (*κ*) was obtained using the equation *κ* = *DρC*_p_, where *D is* the thermal diffusivity, *ρ* is the bulk density measured by the Archimedes method, and *C*_p_ is the specific heat capacity obtained by the Dulong–Petit approximation. Hall effect measurements of carrier concentration (*p*_H_) and mobility (*µ*_H_) on square-shape samples were performed in a van der Pauw configuration by Ecopia (HMS-3000) system at room temperature.

## 3. Results and Discussion

**Figure 1:**
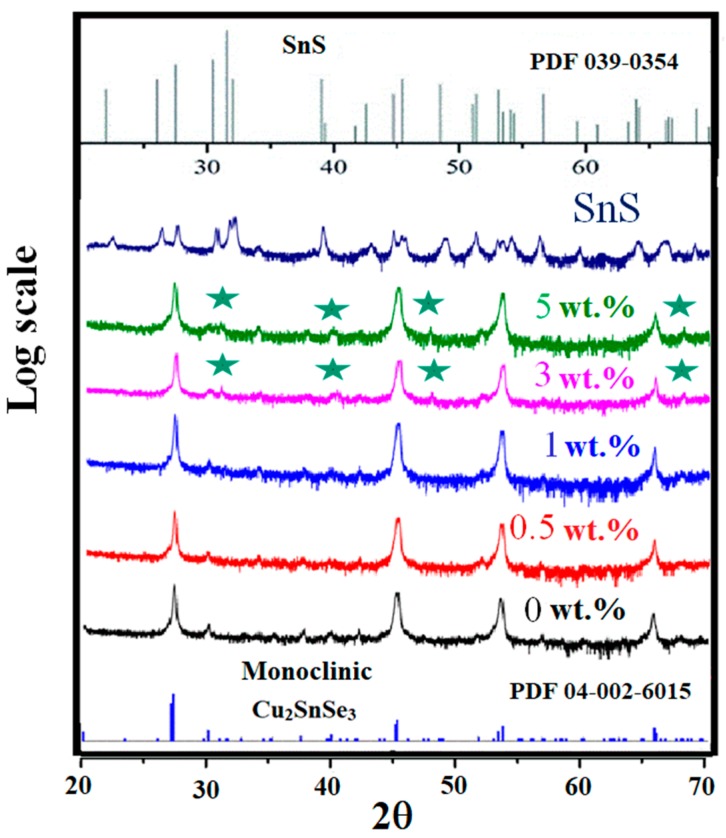
X-ray diffraction (XRD) patterns of Cu_2_SnSe_3_-SnS composite samples spark plasma sintered at 670 K. The bottom and top spectra represent the XRD patterns of CTSe and SnS, respectively, while the other spectra show those of composite samples. Minor peaks indicated by stars in the patterns of 3 and 5 wt% samples correspond to the SnS phase.

**Figure 2:**
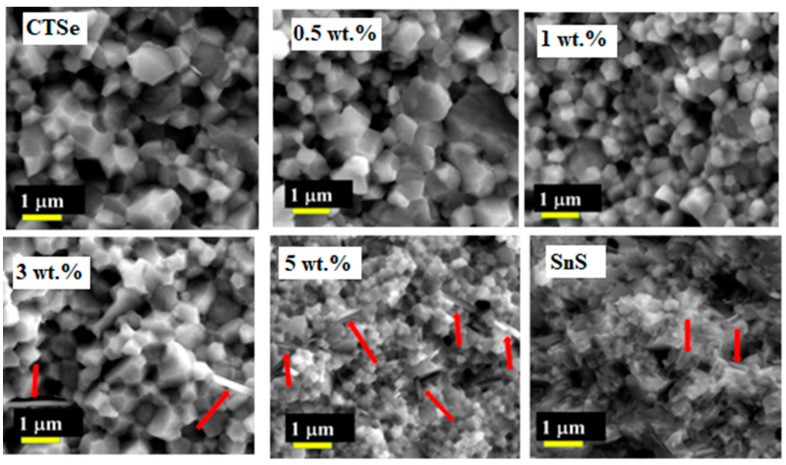
Field emission-scanning electron microscope (FE-SEM) images of the Cu_2_SnSe_3_, SnS, and Cu_2_SnSe_3_-SnS composite samples. The FE-SEM analyses were performed on non-polished surfaces of broken pellets. Red arrows indicate the SnS secondary phase within the CTSe matrix.

Figure 1 shows the XRD patterns of CTSe, SnS, and CTSe-SnS composites, recorded in the range of 2θ from 20 to 70 degree. CTSe and composite samples have the monoclinic phase structure. The patterns of 3 and 5 wt.% CTSe-SnS composites have peaks with very small intensities between 30 and 45 degrees 2θ, most of which come from the SnS phase. Figure 2 shows FE-SEM images of fracture surfaces of the CTSe, SnS, and CTSe-SnS composite samples. Red arrows indicate the SnS phase. The images show that the grain size is decreased with the increase of the amount of SnS in the CTSe matrix. To further investigate the effect of SnS addition to CTSe on the crystallite size, we analyzed the average crystallite size and micro strain by Williamson and Hall (W-H) method [14]. 

**Figure 3:**
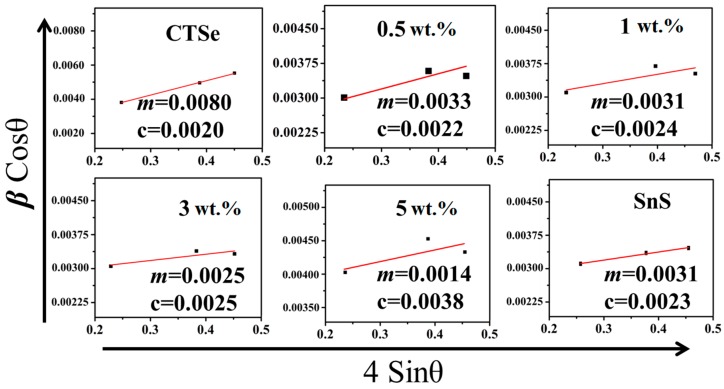
Results of Williamson and Hall (W-H) method. Plot of β_hkl_ cosθ vs. 4 sinθ of the spark plasma sintered Cu_2_SnSe_3_, SnS, and Cu_2_SnSe_3_-SnS composites.

The uniform distribution model (UDM) was applied in W-H method, where it is assumed that the strain is same for all crystallographic directions in the crystal [15]. The modeled equation for the UDM in W-H method consists of both size and strain factors, represented by [15]:

(1) $$\beta_{hkl}\;\cos\theta \;=\;\frac{K\lambda }{D}\;+\;4\varepsilon \sin\theta$$

where β_hkl_ represents the full width at half maximum (FWHM) of a radiant peak. K is crystallite shape constant (0.9), λ is the wavelength of X-ray in nanometer (nm), and ε is the strain. By plotting 4εsinθ on x-axis with β*_hkl_*cosθ on y-axis, the strain of the crystallites was estimated from the slope of the line, while the average size was calculated from the y-axis intercept. The W-H plots for the studied samples are given in Figure 3, while the extracted data is provided in Table 1. We also estimated the average size of the crystallites for studied samples using the Scherrer’s formula (Table 1). The sizes of the crystallites obtained from the Scherrer’s formula and the W-H method which are known to be semi-quantitative methods need to be considered carefully. The absolute numbers (size of crystallites) obtained from those two methods can be different from the numbers (size of grains) which can be obtained from the direct observation (e.g., SEM). Furthermore, the instrument contribution to the breadth of the XRD peaks was not considered in Scherrer’s formula and W-H method because the contribution was identical in all the patterns collected using the same instrument with the same setting. However, the trend of changes can still be compared with those obtained from the direct SEM observation.

**Figure 4:**
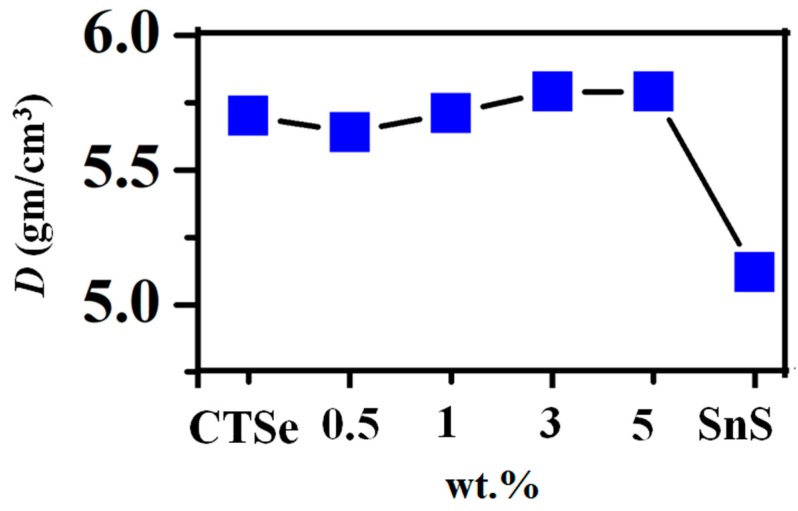
Density of the spark plasma sintered Cu_2_SnSe_3_, SnS, and Cu_2_SnSe_3_-SnS composites. Each point represents the average of five different readings.

**Figure 5:**
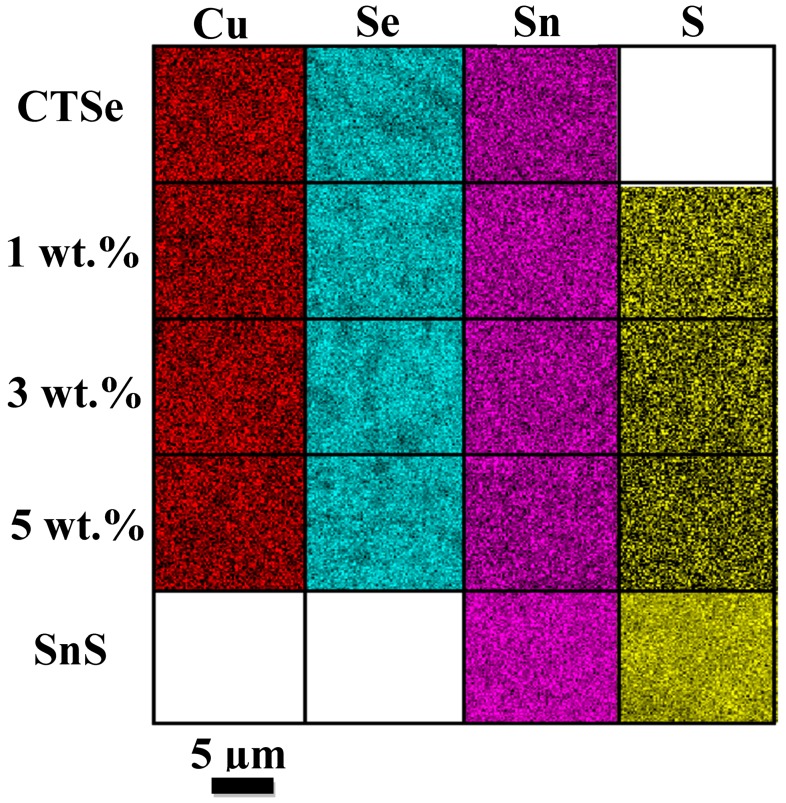
Energy dispersive spectroscopy (EDS) mapping images of the CTSe and SnS composites. The analyses were performed on the polished surfaces of the pellets.

As shown in Table 1, the average variation of the crystallite size is consistent with the SEM data (Figure 2), where the size of grains decreases with the increase in the amount of SnS in the composite samples. The crystallite size values obtained by Scherrer’s formula and W-H method are similar, and the trends in both cases are consistent. The decrease in the grain size in 3 wt.% and 5 wt.% can come from the particles of SnS, which can retard the grain growth. The density of the sintered sample is increased with the increase of the SnS amount as shown in Figure 4, which shows the density of the sintered CTSe, SnS, and CTSe-SnS composites. EDS elemental mapping results given in Figure 5 show that the distribution of the element S is uniform and the composite samples have homogeneous composition in the scale shown in the figure.

**Figure 6:**
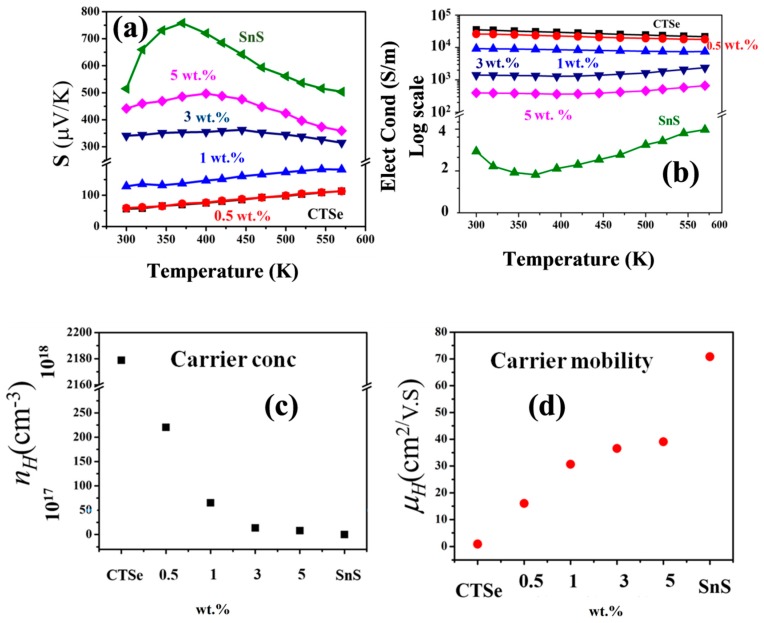
Temperature dependence of (**a**) the Seebeck coefficient, (**b**) electrical conductivity, (**c**) carrier concentration, and (**d**) electronic mobility values of the CTSe, SnS, and CTSe-SnS composites. Each data point of (**c**,**d**) is the average of ten readings recorded on each sample.

Figure 6 shows temperature dependence of the Seebeck coefficient (Figure 6a), as well as the electrical conductivity (Figure 6b) of spark plasma sintered CTSe, SnS, and composites. The Hall-Effect data measured at room temperature are given in Figure 6c–d and also in Table 2. Positive Seebeck coefficient is recorded for all the samples in the temperature range from 300 to 570 K (Figure 6a), which confirms the *p*-type nature of CTSe, SnS, and CTSe-SnS composites. The Seebeck coefficient value of the pristine CTSe is in the range of 30 to 59 μV/K, which is increased up to 500 μV/K by making composites with SnS. The large increase in Seebeck coefficient of the CTSe-SnS can come from the high Seebeck coefficient of SnS and the increase of carrier mobility. Adding SnS to the CTSe matrix results in the decrease of carrier concentration (Figure 6c) while carrier mobility is increased (Figure 6d). This can result from the misalignment of energy bands between host and foreign materials. With the misalignment of energy bands, a large potential barrier can block both majority and minority carriers, and only very high-energy carriers can pass through this potential barrier with small probability of scattering. The overall effect leads to low carrier concentration yet with high mobility within the composite samples.

The band misalignment permits the minority carriers to excite in the direction opposite to the majority carriers (*p*-type or *n*-type) so that large voltage is retained while the Seebeck coefficient increases [16,17]. Figure 6a shows that the Seebeck coefficient of CTSe is not only enhanced, but its behavior with increasing temperature is changed by adding a small amount (3 wt.%) of SnS to the CTSe matrix, due to the large decrease in carrier concentration. The behavior of the Seebeck coefficient with increasing temperature of 0.5 and 1 wt.% resembles that of the CTSe sample, while samples with more than 1 wt.% SnS show bipolar behaviors of the Seebeck coefficient, which is similar to that of SnS [18].

**Figure 7:**
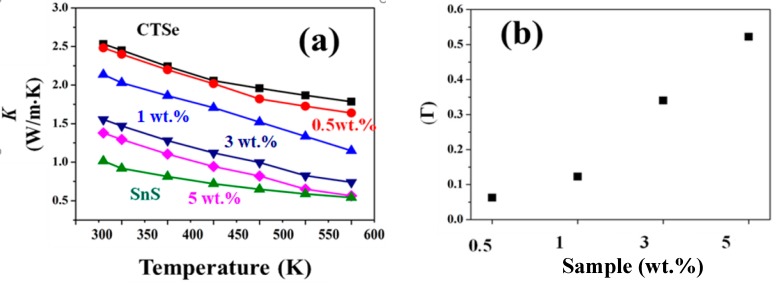
(**a**) Temperature dependence of thermal conductivity of CTSe, SnS, and CTSe-SnS composites and (**b**) lattice scattering parameter values of the CTSe-SnS composite samples.

**Figure 8:**
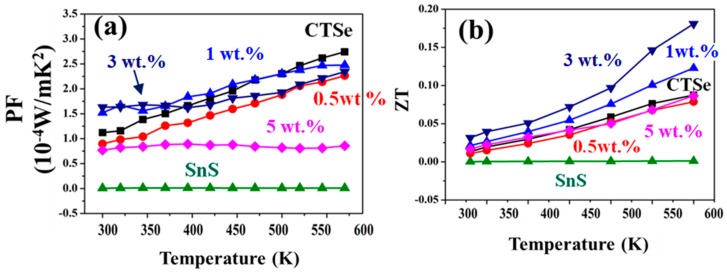
(**a**) Power factor and (**b**) ZT of CTSe, SnS, and Cu_2_SnSe_3_-SnS composites in the temperature range from 300 to 570 K.

Figure 6b shows the temperature dependence of the electrical conductivity from 300–570 K. A decrease of electrical conductivity with increasing temperature of 0.5 and 0 wt.% composite samples reveals the highly doped nature of all the samples. The reduction in the electrical conductivity of the composite samples compared to that of CTSe is in agreement with the carrier concentration data, shown in Figure 6c. As the amount of SnS in the CTSe matrix is increased, the carrier concentration is reduced and electrical conductivity is suppressed. Figure 7a shows the change of thermal conductivity of the CTSe and CTSe-SnS composites at different temperatures. The lattice distortion and defects in the CTSe after incorporation of SnS can reduce the thermal conductivity of the parent matrix, due to the extra disorder scattering (Figure 7b). In contrast, a large improvement of ZT was observed (Figure 8b) due to the reduction of thermal conductivity. 

It should be noted that further addition of SnS to the CTSe matrix in the sample with 5 wt.%, leads to a decrease in both the power factor and ZT, due to the poor electronic transport nature of the sample.

According to Abeles et al. [19], the composite matrix is assumed to have a random distribution of its species in a proper lattice. The resulted scattering of the phonon is larger due to displaced position and anharmonicity of the crystal lattice. One common way to find the degree of disorder scattering of the composites is calculating the scattering parameter (Γ). Large Γ means large phonon scattering, which can decrease the thermal conductivity.

Γ consists of strain induced by point defects, bonding force difference and mass difference for the binary or pseudo-binary alloy systems [20].

(2) $$\Gamma \;=x\cdot y\left[{\left(\frac{\Delta M}{M}\right)}^{2}\;+\;\varepsilon {\left(\frac{\Delta \alpha }{\alpha }\right)}^{2}\right]$$

*M* and *α* are the mean mass and lattice constants of the binary components in the alloy, while Δ*M* and Δ*α* are the differences in molar mass and lattice constants within the virtual crystal, respectively. *ε* in Equation (2) is a fitting parameter, which describes the elastic properties of a lattice system [21]. Numerical value of *ε* can be estimated as:

(3) $$\varepsilon \;=\;\frac{2}{9}{\left[\left(G\;+\;6.4\gamma \right)\frac{1\;+\;\nu }{1\;+\;\nu }\right]}^{2}$$

*G* is a relative constant, which remain the same for the similar compound systems [22,23], *γ* is the Gruneisen parameter, and *ν* is the Poisson ratio. We used *γ* = 1.3 and *G* = 4 for the chalcogenide alloy [24,25] to fit the *ε* value which is equal to 94. The values of *γ*, *G,* and *ε* used in this study are in good agreement with those previously reported [26,27]. The results obtained from Equation (2) are plotted for the four composite samples at room temperature in Figure 7b. It shows that increasing the amount of foreign atoms in the parent matrix results in the increase of the scattering parameter. The change of scattering parameter is consistent with the change of thermal conductivity in Figure 7a and results reported before [28,29].

Figure 8 shows the effect of adding different amounts of SnS to CTSe on the power factor and the ZT of the CTSe and composite samples, measured in the temperature range from room temperature to 570 K. Large improvements of the Seebeck coefficient were observed in the composites (Figure 6a), but the change of the power factor was not significant (Figure 8a), which can result from the synergetic effect of the reduction in electrical conductivity (Figure 6b). In contrast, a large improvement of ZT was observed (Figure 8b) due to the reduction of thermal conductivity. The sample with 3 wt.% SnS has peak ZT equal to 0.18, which is comparable with those previously reported [30,31,32]. It should be noted that further addition of SnS to the CTSe matrix in the sample with 5 wt.%, leads to a decrease in both the power factor and ZT due to the poor electronic transport nature of the sample.

## 4. Conclusions

The Cu2SnSe3 (CTSe)-SnS composites were prepared by mechanical-alloying and spark plasma sintering. The effect of adding different amount of SnS to CTSe on the thermoelectric properties of the composite was investigated. The addition of SnS to CTSe was found to improve the Seebeck coefficient, but the change of power factor was not substantial due to the loss of electrical conductivity. Improvement of ZT in the medium temperature range, however, is achieved by adding 3 wt.% SnS to the CTSe matrix, which can be attributed to the disorder scattering and the decrease in the thermal conductivity of the composites compared to that of the pristine CTSe. The results of this study shows that the Seebeck coefficient and thermal conductivity of CTSe can be controlled in the direction of increasing ZT by making a composite with materials that have the Seebeck coefficient higher than CTSe and have no reaction with CTSe at the temperature range used to sinter CTSe. However, the concurrent suppression of electrical conductivity still needs to be controlled to further improve the thermoelectric performance of CTSe.

## Figures and Tables

**Table 1:** Geometric parameters of the spark plasma sintered Cu_2_SnSe_3_, SnS, and Cu_2_SnSe_3_-SnS composites. UDM: uniform distribution model.

Sample	Scherer’s Method	W-H Method	
		UDM	
	*D* (nm)	*D* (nm)	ε (10^−3^)
CTSe	49.21	67.97	8.03
0.5 wt.%	41.39	62.74	3.29
1 wt.%	40.47	58.50	3.07
3 wt.%	38.42	55.91	2.47
5 wt.%	32.14	36.30	1.41
SnS	34.62	60.02	3.46

**Table 2:** Hall-Effect data of the spark plasma sintered Cu_2_SnSe_3_, SnS, and Cu_2_SnSe_3_-SnS composites.

Sample	Carrier Conc	Carrier Mobility
	*n*_H_ (cm^−3^) (10^17^)	*μ*_H_ (cm^2^/V·S)
CTSe	21788.21	0.89
0.5 wt.%	220.35	16.03
1 wt.%	65.15	30.67
3 wt.%	13.58	36.56
5 wt.%	0.80	39.05
SnS	0.02	73.23

## References

[1] Bell L.E. (2008). Cooling, Heating, Generating Power, and Recovering Waste Heat with Thermoelectric Systems. Science.

[2] Minnich A., Dresselhaus M.S., Ren Z.F., Chen G. (2009). Bulk Nanostructured Thermoelectric Materials: Current Research and Future Prospects. Energy Environ. Sci..

[3] Park D., Ju H., Oh T., Kim J. (2018). Facile Fabrication of One-Dimensional Te/Cu_2_Te Nanorod Composites with Improved Thermoelectric Power Factor and Low Thermal Conductivity. Sci. Rep..

[4] Biswas K., He J., Blum I.D., Wu C.I., Hogan T.P., Seidman D.N., Dravid V.P., Kanatzidis M.G. (2012). High-Performance Bulk Thermoelectrics with All-Scale Hierarchical Architectures. Nature.

[5] Mehta R.J., Zhang Y., Karthik C., Singh B., Siegel R.W., Borca-Tasciuc T., Ramanath G. (2012). A New Class of Doped Nanobulk High-Figure-of-Merit Thermoelectrics by Scalable Bottom-up Assembly. Nat. Mater..

[6] Zhao L., Fei F.Y., Wang J., Wang F., Wang C., Li J., Wang Y., Cheng Z., Dou S., Wang X. (2017). Improvement of Thermoelectric Properties and Their Correlations with Electron Effective Mass in Cu_1.98_S_x_Se_1−X_. Sci. Rep..

[7] Chauhan N.S., Bhardwaj A., Senguttuvan T.D., Pant R.P., Mallikd R.C., Misra D.K. (2016). A Synergistic Combination of Atomic Scale Structural Engineering and Panoscopic Approach in P-Type Zrcosb-Based Half-Heusler Thermoelectric Materials for Achieving High Zt. J. Mater. Chem. C.

[8] Zhang C.H., Ng H., Li Z., Khor K.A., Xiong Q.H. (2017). Minority Carrier Blocking to Enhance the Thermoelectric Performance of Solution-Processed Bi_x_Sb_2-X_Te_3_ Nanocomposites Via a Liquid-Phase Sintering Process. ACS Appl. Mater. Interfaces.

[9] Song D.X., Ma W.G., Zhang X. (2019). Lattice Thermal Conductivity of Si/Ge Composite Thermoelectric Material: Effect of Si Particle Distribution. Int. J. Energy Res..

[10] Pei Y.Z., LaLonde A.D., Heinz N.A., Snyder G.J. (2012). High Thermoelectric Figure of Merit in PbTe Alloys Demonstrated in PbTe-CdTe. Adv. Energy Mater..

[11] Pei Y.Z., Gibbs Z.M., Gloskovskii A., Balke B., Zeier W.G., Snyder G.J. (2014). Optimum Carrier Concentration in N-Type PbTe Thermoelectrics. Adv. Energy Mater..

[12] Byeon D., Sobota R., Delime-Codrin K., Choi S., Hirata K., Adachi M., Kiyama M., Matsuura T., Yamamoto Y., Matsunami M. (2019). Discovery of Colossal Seebeck Effect in Metallic Cu_2_Se. Nat. Commun..

[13] Liu Y., Zhao L.D., Liu Y.C., Lan J.L., Xu W., Li F., Zhang B.P., Berardan D., Dragoe N., Lin Y.H. (2011). Remarkable Enhancement in Thermoelectric Performance of BiCuSeO by Cu Deficiencies. J. Am. Chem. Soc..

[14] Williamson G.K., Hall W.H. (1953). X-ray line broadening from filed aluminium and wolfram. Acta Metall..

[15] Aly K.A., Khalil N.M., Algamal Y., Saleem Q.M.A. (2017). Estimation of Lattice Strain for Zirconia Nano-Particles Based on Williamson-Hall Analysis. Mater. Chem. Phys..

[16] Banik A., Vishal B., Perumal S., Datta R., Biswas K. (2016). The Origin of Low Thermal Conductivity in Sn_1−X_Sb_x_Te: Phonon Scattering Via Layered Intergrowth Nanostructures. Energy Environ. Sci..

[17] Hobbs C.C., Fonseca L.R.C., Knizhnik A., Dhandapani V., Samavedam S.B., Taylor W.J., Grant J.M., Dip L.G., Triyoso D.H., Hegde R.I. (2004). Fermi-Level Pinning at the Polysilicon/Metal Oxide Interface—Part I. IEEE Trans. Electron Dev..

[18] Wang C., Chen Y.D., Jiang J., Zhang R., Niu Y., Zhou T., Xia J.F., Tian H.Q., Hu J., Yang P. (2017). Improved Thermoelectric Properties of SnS Synthesized by Chemical Precipitation. RSC Adv..

[19] Abeles B. (1963). Lattice Thermal Conductivity of Disordered Semiconductor Alloys at High Temperatures. Phys. Rev..

[20] Alekseeva G.T., Efimova B.A., Ostrovskaya L.M., Serebryannikova O.S., Tsypin M.I. (1971). Thermal conductivity of solid solutions based on lead telluride. Sov. Phys. Semicond..

[21] Wang H., LaLonde A.D., Pei Y.Z., Snyder G.J. (2013). The Criteria for Beneficial Disorder in Thermoelectric Solid Solutions. Adv. Funct. Mater..

[22] Wang H., Wang J.L., Cao X.L., Snyder G.J. (2014). Thermoelectric Alloys between PbSe and PbS with Effective Thermal Conductivity Reduction and High Figure of Merit. J. Mater. Chem. A.

[23] Jin H.Y., Jaworski C.M., Heremans J.P. (2012). Enhancement in the Figure of Merit of P-Type Bi_100–X_Sb_x_ Alloys through Multiple Valence-Band Doping. Appl. Phys. Lett..

[24] Anderson O.L., Nafe J.E. (1965). The Bulk Modulus-Volume Relationship for Oxide Compounds and Related Geophysical Problems. J. Geophys. Res..

[25] Anderson D.L., Anderson O.L. (1970). The Bulk Modulus Volume Relationship for Oxides. J. Geophys. Res..

[26] Callaway J., von Baeyer H.C. (1960). Effect of Point Imperfections on Lattice Thermal Conductivity. Phys. Rev..

[27] Klemens P.G. (1960). Thermal Resistance Due to Point Defects at High Temperatures. Phys. Rev..

[28] Qiu P.F., Huang X.Y., Chen X.H., Chen L.D. (2009). Enhanced Thermoelectric Performance by the Combination of Alloying and Doping in TiCoSb-Based Half-Heusler Compounds. J. Appl. Phys..

[29] Pei Y.Z., Wang H., Snyder G.J. (2012). Band Engineering of Thermoelectric Materials. Adv. Mater..

[30] Fan J., Schnelle W., Antonyshyn I., Veremchuk I., Carrillo-Cabrera W., Shi X., Grin Y., Chen L.D. (2014). Structural Evolvement and Thermoelectric Properties of Cu_3−X_Sn_x_Se_3_ Compounds with Diamond-Like Crystal Structures. Dalton Trans..

[31] Li Y.Y., Liu G.H., Cao T.F., Liu L.M., Li J.T., Chen K.X., Li L.F., Han Y.M., Zhou M. (2016). Enhanced Thermoelectric Properties of Cu_2_SnSe_3_ by (Ag, In)-Co-Doping. Adv. Funct. Mater..

[32] Shi X.Y., Xi L.L., Fan J., Zhang W.Q., Chen L.D. (2010). Cu-Se Bond Network and Thermoelectric Compounds with Complex Diamondlike Structure. Chem. Mater..

